# Ticks and Tick-Borne Pathogens in Wild Animals and Birds from Two Rehabilitation Facilities in Greece

**DOI:** 10.3390/pathogens14010009

**Published:** 2024-12-28

**Authors:** Dimitrios Vourvidis, Georgia Tzouganatou, Sokratis Perdikaris, Evangelia Kofidou, Beatriz Martinez-Gonzalez, Mary Emmanouil, Emmanouil Papadogiannakis, Anastasia Komnenou, Emmanouil Angelakis

**Affiliations:** 1 Diagnostic Department and Public Health Laboratories, Hellenic Pasteur Institute, 11521 Athens, Greece; dvourvidis@gmail.com (D.V.); g.tzouganatou@pasteur.gr (G.T.); socper@gmail.com (S.P.); bmartinez@pasteur.gr (B.M.-G.); emmanouilm@pasteur.gr (M.E.); 2 Directorate of Veterinary Laboratory Center, Ministry of Rural Development and Food, 11521 Athens, Greece; 3 Directorate of Animal Health, General Directorate of Veterinary Services, Ministry of Rural Development and Food, 11521 Athens, Greece; 4 School of Veterinary Medicine, Faculty of Health Sciences, Aristotle University of Thessaloniki, 54124 Thessaloniki, Greece; kofidouen@vet.auth.gr (E.K.); natakomn@vet.auth.gr (A.K.); 5 Department of Public Health Policy, School of Public Health, University of West Attica, 11521 Athens, Greece; mpapadogiannakis@uniwa.gr

**Keywords:** tick-borne pathogens, wildlife, Greece, *Rickettsia* spp., *Anaplasma* spp., parasites, molecular techniques, Sanger sequencing

## Abstract

Ticks are temporary ectoparasites that serve as vectors for a wide range of pathogens affecting both wildlife and humans. In Greece, research on the prevalence of tick-borne pathogens in wildlife is limited. This study investigates the presence of pathogens, including *Anaplasma* spp., *Babesia* spp., *Bartonella* spp., *Rickettsia* spp., as well as tick-borne encephalitis (TBE), and Crimean–Congo hemorrhagic fever (CCHF) viruses, in ticks collected from 28 wild animals across 10 different animal species. Ticks were manually harvested and identified using molecular methods, with sequencing confirming the presence of *Hyalomma aegyptium*, *H. marginatum*, *H. anatolicum*, *Ixodes frontalis*, and *I. ventalloi*. Among the pathogens detected, *R. aeschlimannii* was the most prevalent, particularly in *H. aegyptium* ticks from tortoises. Additionally, *R. africae* was identified in *H. aegyptium* from tortoises, marking the first report of this pathogen in this tick species in Greece. *Hemolivia mauritanica*, an apicomplexan parasite commonly found in *Testudo* tortoises, was also detected. No evidence of *Babesia* spp., *Anaplasma* spp., *Bartonella* spp., or viral nucleic acid was found. Phylogenetic analysis revealed close genetic relationships between the detected *Rickettsia* species and those previously reported in neighboring regions. These findings underscore the role of wildlife in the epidemiology of tick-borne pathogens in Greece and highlight the need for comprehensive surveillance to prevent future outbreaks.

## 1. Introduction

Ticks are temporary obligate ectoparasites of vertebrates and serve as vectors for a diverse array of pathogens, constituting one of the most frequent vectors affecting a wide range of vertebrate hosts, from wildlife to humans [1]. Ticks serve not only as vectors but also as active environments for tick-borne pathogens, where interactions with the tick microbiome can potentially alter pathogen virulence, infectivity, and transmission dynamics. Small wildlife species play a crucial role as reservoir hosts in the enzootic cycle of tick-borne pathogens, facilitating their transmission to humans and domestic or companion animals through tick vectors. For instance, wild boars have been identified as reservoir hosts for *Anaplasma phagocytophilum* in Europe [2]. Recent increases in global temperatures have enhanced tick survival rates, thereby expanding their geographic range, as evidenced by the spread of the *Hyalomma* genus throughout the Balkan region. This evolutionary adaptation, combined with their expanded distribution, can drive the emergence of new pathogen strains, posing increased risks to both wildlife and human populations.

The evolution of hematophagy in arthropods is closely linked to early reptiles which can harbor a variety of zoonotic pathogens and may serve as blood meal sources for vectors that also feed on humans, thereby connecting reptilian and human disease transmission cycles. Ticks are well-adapted to reptilian hosts, often causing minimal harm while transmitting pathogens such as *Anaplasma* and *Rickettsia*, both of which include reptilian-associated clades, although their reservoir role is still unclear [3]. Tortoises from the *Testudo* genus are known hosts of ticks from the *Hyalomma* genus, with infectious agents such as *Rickettsia* spp., *Ehrlichia* spp., *Anaplasma* spp., and *Hemolivia* spp., detected in Greece and other European countries such as Italy [4] and Anatolia [5]. Hedgehogs, on the other hand, exhibit a high prevalence of ectoparasites [6] and often experience significant infestation intensities [7]. Previous studies on European and Northern white-breasted hedgehogs have identified DNA from *Anaplasma phagocytophilum* [8], *R. helvetica* [9], *Bartonella* spp., and *Babesia* spp. [10]. Wild birds are frequent tick hosts and can transport ticks over long distances via migration, facilitating the spread of tick-borne pathogens. In Greece, data on wild birds remain limited, with only two studies—one on Antikythira Island and another in Northern Greece—reporting the detection of *Rickettsia* spp. and *R. aeschlimannii* [11]. However, in Italy, Spain, Hungary [12], Germany [13], Poland [14], and Romania [15] ticks collected from wild birds have been found to carry bacterial pathogens such as *Anaplasma* spp. and *Rickettsia* spp. [16], as well as parasitic agent *Babesia* spp.

Tick-borne pathogens pose significant public health risks in Greece, with various species identified in recent studies. Spotted fever group *Rickettsia* (e.g., *R. massiliae*, *R. slovaca*, *R. raoulti*, *R. hoogstraalii*, *R. monacensis*) has been detected in ticks collected from sheep, goats and dogs in Northern and Central Greece. Additionally, *R. aeschlimannii* has been found in ticks collected from wild birds on Antikythira island. Other pathogens, such as *Anaplasma* spp., and *Babesia* spp., are prevalent in *I. ricinus* ticks from goats and sheep in Northern Greece while *Ehrlichia* spp. has been detected in *R. turanicus* ticks from sheep on Lesvos island. Environmental factors, including temperature, rainfall, and altitude significantly influence the distribution of these pathogens [17]. Despite the growing concern, most research focuses on ticks as vectors for domestic animals or humans, with limited data available on their distribution among Greece’s wildlife [18], even though similar studies have been conducted in other European countries as Italy [19], France [20] and Romania [21]. Therefore, it is crucial to investigate the extent to which wild animals contribute to the enzootic cycle of tick-borne pathogens, and to elucidate the transmission mechanisms involving wild animals and *Ixodid* tick species [22].

Consequently, due to the limited molecular epidemiological data on tick-borne pathogens in Greece’s wildlife, our study aimed to investigate bacterial infections caused by *Anaplasma* spp., *Babesia* spp., *Bartonella* spp., and *Rickettsia* spp., as well as viral infections including tick-borne encephalitis (TBE) and Crimean–Congo hemorrhagic fever (CCHF) in ticks collected from wild animals admitted for treatment in two rehabilitation facilities.

## 2. Materials and Methods

### 2.1. Study Area and Tick Collection

**Figure 1:**
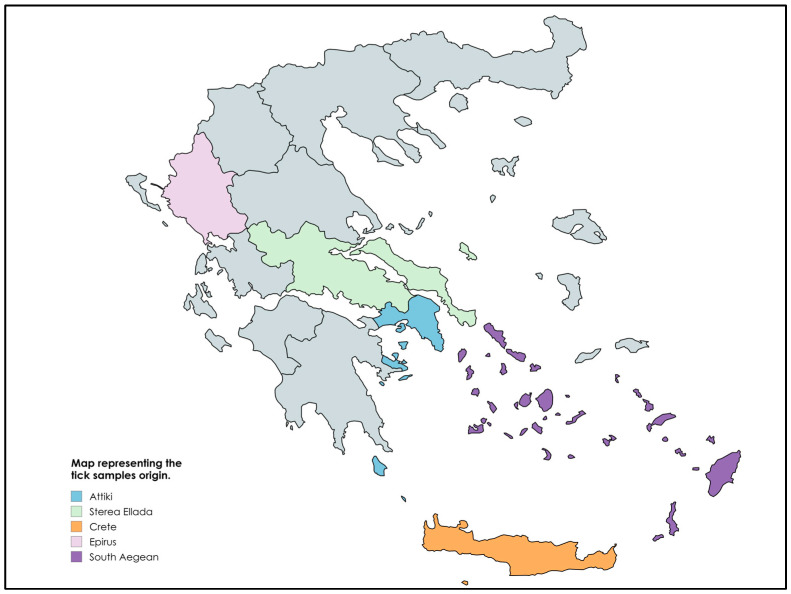
Map representing the origin of the tick samples.

A total of 81 live feeding ticks were collected from 28 wild animals during the spring and summer of 2024 across Greece, including 46 ticks from 19 tortoises *Testudo marginata*, 18 ticks from one barn owl *Tyto alba*, one tick from one stock pigeon *Columba oenas*, two ticks from one hedgehog *Erinaceus europaeus*, one tick from one little owl *Athene noctua*, two ticks from one Mediterranean tortoise *Testudo hermanni*, six ticks were collected from one common buzzard *Buteo buteo*, one tick from one Eurasian magpie *Pica pica*, one tick from one fox *Canis vulpes*, and three ticks from one European brown hare *Lepus europaeus*. The wild animals from which the ticks were harvested were admitted for treatment to the “Exotic and Wildlife Department” of the School of Veterinary Medicine, Aristotle University of Thessaloniki, and to the “Wildlife Rehabilitation Center-ANIMA” in several parts of Greece. The origin of the samples is presented in Figure 1, which was created using mapChart (https://www.mapchart.net/, accessed on 1 November 2024) . All the wild animals admitted for rehabilitation for various causes (e.g., injuries, dehydration, emaciation etc.) were recorded in an electronic database along with all the accompanying information and history, including the animal’s age, sex, and region of origin. Ticks were collected manually on animal presentation after stabilization of its general condition. They were subsequently stored in deep freeze −20 °C and then were transferred to the Hellenic Pasteur Institute in Athens, where they were stored at −80 °C until further analysis.

### 2.2. Nucleic Acid Extraction

Nucleic acid extraction was performed on individual tick samples. Ticks were first washed twice with sterile distilled water and air-dried to remove impurities and contaminations before homogenization. Once dried, each tick was cut vertically into two equal halves using sterile scalpels, and then manually homogenized with sterile pestles. The homogenized tick samples were digested with proteinase K and lysis buffer overnight at 56 °C. Total DNA and RNA were extracted using an automated extraction kit (MagCore, RBC Bioscience New Taipei city, Taiwan), using the MagCore^®^ Genomic DNA Tissue Kit and MagCore^®^ Viral Nucleic Acid Extraction Kit (High Sensitivity), according to the recommendations of the manufacturer. After the nucleic acid extraction, all samples were quantified using a spectrophotometer in order to assess and assure the quantity and purity of the nucleic acid yielded. All extracted samples were stored at −80 °C until further analysis. Each tick was processed and screened individually using real-time PCR and sequencing to identify tick species and detect apicomplexan parasites, bacterial, and viral pathogens.

### 2.3. PCR Amplification

For molecular identification of the tick species, the extracted tick DNA was subjected to conventional PCR amplification and Sanger sequencing based on the *12S rDNA* sequence (Table 1) [23]. Additionally, the extracted DNA was subjected to real time PCR analysis using specific primer and probe sequences designed to detect conserved regions of genes to detect the presence of *Anaplasma*/*Ehrlichia* spp. [24], *Rickettsia* spp. [25], *Babesia* spp. [26], and *Bartonella* spp. [25] at the genus level (Table 1). RNA samples were analyzed by PCR to detect the presence of CCHF [27] and TBE [28] viruses. Each PCR amplification included both negative and positive controls specific to the target pathogens. For *Anaplasma* spp., *A. phagocytophilum* was used as the positive control; for *Bartonella* spp., *B. quintana* served as the positive control; for *Rickettsia* spp., *R. conorii* was employed as the positive control; and for *Babesia* spp., *B. microti* was included as the positive control, which also served for the amplification of apicomplexan parasites. For the detection of viruses, positive controls of CCHF and TBE were utilized, respectively.

### 2.4. Sequence and Phylogenetic Analysis

All tick DNA samples and positive PCR products were amplified using conventional PCR targeting different genetic markers. *Rickettsia* spp. were detected by amplifying a partial sequence of the 639 bp of outer membrane protein A (*ompA*) gene [29]. Detection of apicomplexan parasites was achieved using specific primers targeting a partial *18S rRNA* gene (Table 1) [30]. PCR products were visualized using the Qiagen QIAxcel Advanced system (Qiagen, Hilden, Germany). Amplified PCR products of the expected size were purified with the QIAquick PCR purification kit (Qiagen, Hilden, Germany) and subsequent Sanger sequencing was performed on the purified amplicons using the corresponding forward and reverse primers for each pathogen. Sequence alignment was performed using Clustal W software version 2.1 (https://www.genome.jp/tools-bin/clustalw/, accessed on 5 August 2024) and the resulting sequences were compared using the Basic Local Alignment Search Tool (BLAST) of the National Center for Biotechnology Information (NCBI) (http://blast.ncbi.nlm.nih.gov/Blast.cgi/, accessed on 5 August 2024).

Phylogenetic analysis of *Rickettsia* spp. and *Hemolivia* spp. samples was performed using the Molecular Evolutionary Genetics Analysis software (MEGA-11). Reference sequences for the *Rickettsia* samples were obtained from the *ompA* gene, while partial *18S rRNA* gene sequences were used for *Hemolivia* samples. All reference sequences were sourced from the National Center for Biotechnology Information (NCBI) database (http://blast.ncbi.nlm.nih.gov/Blast.cgi/, accessed on 20 August 2024) and aligned with the nucleotide sequences from this study using Clustal W software version 2.1 (https://www.genome.jp/tools-bin/clustalw/, accessed on 20 August 2024), followed by manual trimming to ensure uniform length. Phylogenetic trees were constructed using the neighbor-joining method, applying the p-distance model with a non-parametric bootstrap of 1000 replicates, to assess the robustness of the inferred relationships. All positions containing gaps or missing data were excluded (complete deletion).

## 3. Results

### 3.1. Tick Collection Outcomes

Sequencing of the amplified *12S rRNA* gene fragment revealed that the majority of the ticks (79/81, 98%) belonged to the *Hyalomma* genus with 58 out of the 79 (73%) ticks being *Hyalomma aegyptium*, 18 (23%) *H. marginatum* followed by three *H. anatolicum* ticks (4%). Additionally, two ticks were identified as *Ixodes* species: *I. frontalis* (1%) and *I. ventalloi* (1%) (Table 2).

All tick-infested animals carried only one tick species and no co-infestation between different tick species was detected.

### 3.2. Pathogen Detection Results

Among the pathogens detected, *Rickettsia* spp. was the most prevalent, identified in 23 ticks (28%). Sequencing of the *ompA* gene fragment revealed the presence of several *Rickettsia* species: *R. aeschlimannii* was found in 5 ticks collected from four *T. marginata* tortoises, 11 ticks from a *T. alba* barn owl, 2 ticks from a *T. hermanni* Mediterranean tortoise, one tick from *C. oenas* stock pigeon, and 2 ticks from an *E. europaeus* hedgehog. Additionally, *R. africae* was detected in one tick from a *T. marginata* tortoise (Table 2). Sequencing of two ticks from tortoises that tested positive for *Rickettsia* spp. using real time PCR, was not successful due to troubleshooting during Sanger sequencing. In contrast, no DNA was detected for *Bartonella* spp., *Babesia* spp., or *Anaplasma* spp., nor for the viruses CCHF and TBE.

Sequencing of the apicomplexan *18S rRNA* gene fragment revealed *Hemolivia mauritanica* in 5% of the samples. This parasite was detected in 4 ticks collected from 4 *T. marginata* tortoises (Table 2). Overall, 25 out of 81 ticks (31%) tested positive for at least one pathogen, with 23 ticks (32%) harboring a single pathogen and 2 ticks (2%) carrying 2 pathogens. Coinfections of *Rickettsia* spp. and *Hemolivia* spp. were observed exclusively in ticks from *T. marginata* tortoises, while no evidence of coinfections were found in ticks from other wild animals.

### 3.3. Tick Species and Tick-Borne Pathogens on Host Animals

Ultimately, as presented in Table 2, all tortoises were infested with *H. aegyptium* ticks, with identified pathogens including *R. aeschlimanni* (11%), *R. africae* (2%), and *H. mauritanica* (9%). The ticks from the barn owl were identified as *H. marginatum* carrying *R. aeschlimanni* (61%). The single *H. aegyptium* tick from the stock pigeon was infected with *R. aeschlimannii*, as were the two *H. aegyptium* ticks collected from the hedgehog. In contrast, the one *H. aegyptium* tick found on the little owl was not infected by any of the pathogens tested. Additionally, no pathogens were detected in the six *H. aegyptium* ticks removed from the common buzzard, the *I. frontalis* tick from the Eurasian magpie, the *I. ventalloi* tick from the fox or the three *H. anatolicum* ticks from the European brown hare.

### 3.4. Phylogenetic Analysis

**Figure 2:**
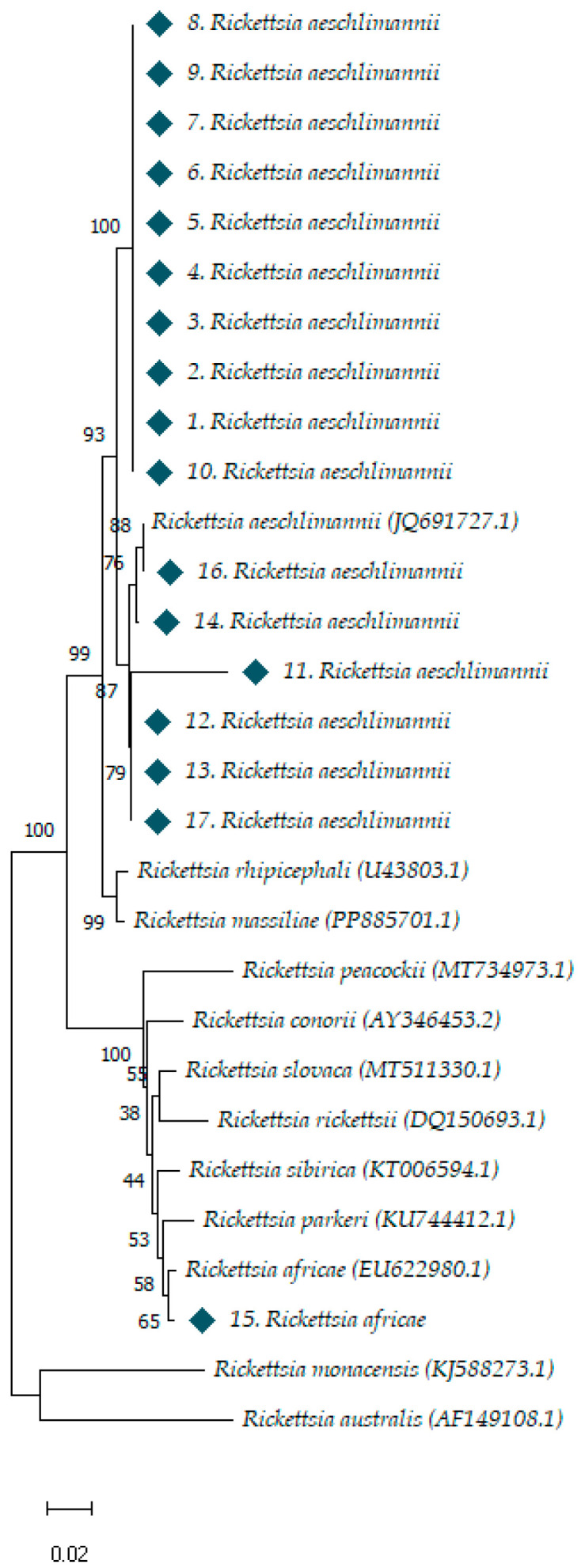
Phylogenetic analysis of Rickettsiae sequences based on *ompA* gene sequences. Analysis was carried out with MEGA-11 software. The sequences were aligned using the alignment program CLUSTAL, which is a part of the MEGA-11 software package. The evolutionary distance values were determined by the method of p-distance and these values were used to construct a phylogenetic tree by the neighbor-joining method. The numbers at nodes are the proportion of 1000 bootstrap that support the topology shown. References sequences of spotted fever *Rickettsia* group were exported from GenBank.

To classify the *Rickettsia* sequences found in ticks, we performed a phylogenetic analysis based on the *ompA* gene sequences from the Spotted Fever *Rickettsia* Group. The phylogenetic analysis, shown in Figure 2, revealed that 16 of our sequences were identical to each other and to *R. aeschlimannii* strain TR/Orkun-H.aegyp85/Ankara (accession number JQ691727) which was obtained from an *H. aegyptium* in Ankara. Additionally, one sequence was identical to *R. africae* (accession number EU622980).

**Figure 3:**
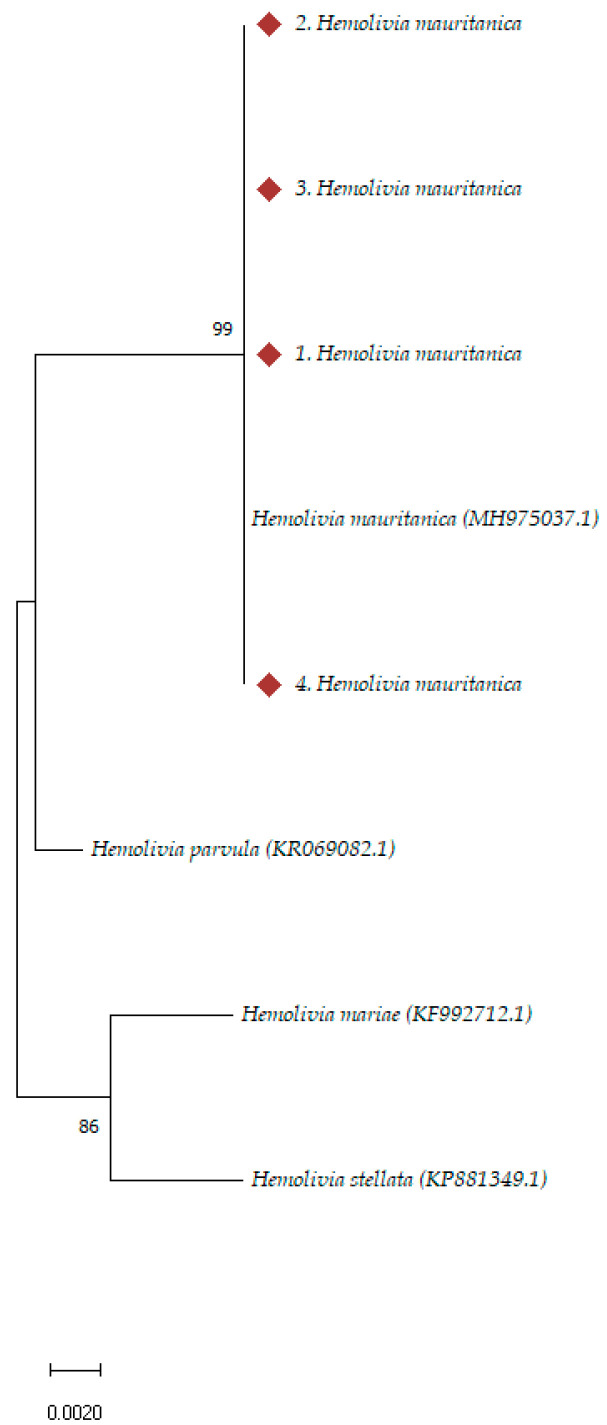
Phylogenetic analysis of Hemolivia positive samples based on *18S rRNA* gene sequences. Analysis was carried out with MEGA-11 software. The sequences were aligned using the alignment program CLUSTAL, which is a part of the MEGA-11 software package. The evolutionary distance values were determined by the method of p-distance and these values were used to construct a phylogenetic tree by the neighbor-joining method. The numbers at nodes are the proportion of 1000 bootstrap that support the topology shown. References sequences of *Hemolivia* spp. were exported from GenBank.

Similarly, the phylogenetic analysis of *Hemolivia* samples was conducted based on *18S rDNA* sequences, using all available *Hemolivia* spp. gene sequences from GenBank. The phylogenetic analysis, presented in Figure 3, indicates that our sequences were identical to each other and to *H. mauritanica* (accession number MH975037.1).

## 4. Discussion

Our results indicate the presence of *Hyalomma* genus (*H. aegyptium*, *H. marginatum*, and *H. anatolicum*) and *Ixodes* genus (*I. frontalis* and *I. ventalloi*) ticks in Greece’s wildlife. These findings align with a previous study on the European continent, which reported *Hyalomma* genus as one of the five main genus in the Balkans alongside *Ixodes*, *Dermacentor*, *Haemophysalis*, and *Rhipicephalus* [31]. Ticks were collected during the spring and summer months, a period when *Hyalomma* species are most abundant, consistent with their seasonal peak in prevalence during these months. Notably, most *Hyalomma* ticks were collected from *Testudo* tortoises, in agreement with Siroky et al., who documented the strong prevalence of all life stages of *H. aegyptium* parasitizing west Palearctic *Testudo* tortoises [32].

*H. aegyptium* ticks were found in six animal species in this study. In Greece, *H. aegyptium* is considered the primary tick vector for *Borrelia* spp. and *Rickettsia* spp. [33]. This tick species has previously been collected from cattle, domestic animals, and humans in Northern Greece as well as from tortoises (*T. marginata* and *T. hermanni*) in Volos, Kardamili, and Sparti [34]. A study conducted in 2020 in European Turkey, which screened *H. aegyptium* ticks for CCHFV, reported that 10% of the samples tested positive for the virus. This finding highlights a potential transmission cycle that could influence the natural dynamics of the virus and its spread to humans [35]. Moreover, ticks from the *Hyalomma* genus (*H. marginatum, H. anatolicum*, and *H. aegyptium)* have also been identified in tortoises and wild birds in Northern Greece. Beyond Greece, *H. aegyptium* has been reported in other countries such as Israel [36] and Turkey [37], where it has been collected from humans, turtles, and hedgehogs [11].

*H. marginatum* ticks were found in all ticks from the barn owl (*T. alba*). Globally, *H. marginatum* is considered the primary arthropod vector for CCHFV. In a study conducted between 2012 and 2013, Chaligiannis et al. reported a prevalence of *H. marginatum* ranging from 0.3% to 17% in ticks collected from sheep, goats, cattle, and humans who had visited hospitals across Greece [38]. It has also been found on Cephalonia island, collected from goats, sheep, cattle, horses, and dogs [39]. *H. marginatum* ticks have also been documented on cattle, birds and humans in numerous European countries including France [40], Austria [41], Spain [42], Italy [43], Bulgaria [44], and Germany [45], where migratory birds are believed to bring *H. marginatum* from distant areas. In the present study, three *H. anatolicum* ticks were removed from a European brown hare *Lepus europaeus*, making the first identification of this tick species on this host. Previously, *H. anatolicum* has been collected from sheep, goats, cattle, and horses in Northern Greece and on Cephalonia island [39,46]. In 2002, a *H. anatolicum* tick was removed from a patient in Crete, who exhibited symptoms of rickettsial infection, including a characteristic skin rash, fever, arthralgia, myalgia, and fatigue. PCR amplification and sequencing of the patient’s blood sample identified *R. sibirica mongolotimonae* as the causative agent [47]. *H. anatolicum* has also been identified in Cyprus [48] in ticks collected from sheep and in Bulgaria [44] in ticks collected from dogs [49].

*I. frontalis* was found in a tick collected from a Eurasian magpie (*Pica pica*). It is an ornithophilic tick poorly studied due to *I. ricinus* being the most important vector to humans and due to its lack of veterinary interest. It is associated with birds and it is known to carry zoonotic pathogens such as *Anaplasma phagocytophilum*, *Rickettsia* spp., and *Borrelia* spp. [50]. In Greece, it has been previously identified in *Turdus merula*, *Fringilla coelebs*, and *Erithacus rubecula* birds of Northern Greece [46]. It has also been found on migratory birds in Russia [51], Italy [52], and Georgia [53]. *I. ventalloi* was collected from one tick vectoring a *Canis vulpes*. It typically infests the European rabbit (*Oryctolagus cuniculus*) but can also be found on other hosts, including birds, mammals, rodents, carnivores, reptiles, and in some cases humans [54]. *Anaplasma* spp., *Rickettsia* spp., *Bartonella* spp., and *Ehrlichia* spp., are among the pathogens that have been associated with *I. ventalloi* with infected ticks collected from wild animals, domestic cats, and even a human, suggesting its potential role as a vector with both medical and veterinary significance [55]. It has also been found in Italy [56] and Portugal [57] on questing ticks. To our knowledge, this is the first instance of *I. ventalloi* tick identification in Greece.

*R. aeschlimannii* was found to be the most common agent, as it was identified in *H. aegyptium* ticks. The first identification of *R. aeschlimannii* in Greece dates back to 2006, when it was found in *H. anatolicum excavatum* ticks removed from a sheep on the island of Cephalonia [39]. Since then, it has been detected with high prevalence in *H. marginatum rufipes* ticks from migratory birds on Antikythira island, in *Rh. sanguineus* and *Rh. turanicus* ticks from domestic animals in Northern Greece and in *H. aegyptium* ticks removed from *Turdus merula* in Northern Greece [11]. However, the first case of *R. aeschlimannii* human infection in Greece was reported much later, in 2013, on Crete Island. The tick removed from the patient was *Rh. Turanicus*, and the patient presented with a typical reddish, painless eschar on his arm, with no other symptoms reported. The identification of the pathogen was made using molecular methods, specifically sequencing fragments of the *gltA* and *ompA* genes [58]. *R. aeschlimannii* has also been found on *H. aegyptium* ticks from the genus *Testudo*, in other countries such as Algeria and on *H. marginatum* ticks from Austria [59], Italy and Germany [45].

*R. africae* infection was detected in a *H. aegyptium* tick collected from *T. marginata*. In Greece, *R. africae* was first reported in 2010 when it was identified from *Rh. sanguineus* ticks removed from humans in the northeast of Greece. It was later detected in *H. marginatum* ticks collected from migratory birds on Antikythira island in 2014. To our knowledge, there has been no further report of *R. africae* infection in humans in Greece, and this is the first report of the agent in *H. aegyptium* ticks in the country. *R. africae* has also been found in *H. aegyptium* ticks collected from *T. marginata* in Israel and Anatolia [36]. *R. africae* is the causative agent of African tick-bite fever, and it is believed to be transmitted by migratory birds that reach Greece, having traversed Africa [60]. The disease caused by this pathogen resembles other tick-bite rickettsioses and is characterized by symptoms such as fever, headaches, myalgia, and multiple eschars [61].

*Hemolivia mauritanica* was identified in 11 ticks collected from *T. marginata*. This parasite, belonging to the phylum *Apicomplexa*, uses reptiles as intermediate hosts and *Ixodid* ticks as its definitive host. In the Mediterranean Rim, it has only been detected in tortoises of the genus *Testudo*, specifically in *H. aegyptium* ticks. It has previously been reported in Greece with a high prevalence of 81% among *T. marginata*, *T. graeca*, and *T. hermanni* tortoises [32]. Recently, *H. mauritanica* was found to be the most prevalent pathogen detected in *H. aegyptium* ticks in Qatar. However, data on this parasite in ticks from Greece remains limited, largely due to its preference for reptiles [62]. Even though we did not detect *Bartonella* spp., *Babesia* spp., and *Anaplasma* spp. in our samples, this does not exclude their presence in Greece, especially in light of recent studies indicating their prevalence in this region [46]. This study represents a small component of a larger research initiative, and further investigations are ongoing, which will provide additional results in the future.

## 5. Conclusions

Wild animals and birds can serve as reservoirs for zoonotic diseases, posing significant public health risks. This study identified tick species and tick-borne pathogens in a community of Greek wild animals and birds, finding five tick species, with *H. aegyptium* and *H. marginatum* being the most common. Notably, *H. anatolicum* was identified in a European brown hare for the first time in Greece, and *I. ventalloi* was also detected for the first time in the country.

The high prevalence of *Rickettsia* species in ticks from various wild animals and birds suggests a high level of endemicity, presenting potential risks for both animals and humans. *R. africae* was detected for the first time in *H. aegyptium* ticks from tortoises, indicating a potential widespread presence in Greece, though further studies are needed to confirm this. The role of certain *Ixodes* species, such as *I. ventalloi* and *I. frontalis*, as vectors remain poorly understood, and their potential for pathogen transmission could pose unrecognized risks if not further investigated. Despite the increasing tick populations and the looming threat of emerging tick-borne pathogens, Greece lacks a national surveillance program, unlike other European countries.

Our results contribute to the current understanding of tick species and tick-borne pathogens associated with wildlife in Greece. Future, more comprehensive research, including the examination of a larger number of ticks from a broader range of wild animal hosts, will help clarify the potential impact of ticks from wildlife on the epidemiology of tick-borne pathogens in the country.

## Figures and Tables

**Table 1:** List of primers and probes used for molecular tick characterization and for detection of infection in ticks.

Organism	Target Gene	Molecular Diagnostic Method	Primers and Probe	Reference
Ticks	*12S rRNA*	Conventional PCR	5′ AAACTAGGATTAGATACCCT 3′ 5′ AATGAGAGCGACGGGCGATGT 3′	[11]
*Anaplasma*/*Ehrlichia* spp.	*23S rRNA*	Real-time PCR	5′ TGACAGCGTACCTTTTGCAT 3′ 5′ TGACAGGTAACAGGTTCGGTCCTCA 3′ 5′ FAM-GGATTAGACCCGAAACCAAG-BHQ1 3′	[12]
*Bartonella* spp.	*gtlA*	Real-time PCR	5′ GATGCCGGGGAAGGTTTTC 3′ 5′ GCCTGGGAGGACTTGAACCT 3′ 5′ FAM-CGCGCGCTTGATAAGCGTGA-BHQ1 3′	[13]
*Rickettsia* spp.	*gtlA*	Real-time PCR	5′ GTGAATGAAAGATTACACTATTTAT 3′ 5′ GTATCTTAGCAATCATTCTAATAGC 3′ 5′ FAM-CGGCAGGTAAGKATGCTACTCAAGATAA-BHQ1 3′	[13]
*Babesia* spp.	*18S rRNA*	Real-time PCR	5′ TTGGGGGCATTCGTANTRAC 3′ 5′ TTCTTGATTAATGAAAACGTCTTG 3′ 5′ FAM-AAGACGAACTACTGCGAAAGCATTTGC-BHQ1 3′	[14]
*CCHF*	*M segment*	Real-time PCR	5′ CAAAGAAACACGTGCCGCTT 3′ 5′ ATTCTCCTCGATTTTGTTTTCCAT 3′ 5′ FAM-ACGCCCACA[BHQ1dT]GTGTTCTCTTGAGTGTTAGCA-BHQ1 3′	[15]
*TBE*	*NS1 protein* *region*	Real-time PCR	5′ TGGAYTTYAGACAGGAAYCAACACA 3′ 5′ TCCAGAGACTYTGRTCDGTGTGGA 3′ 5′-FAMCCCATCACTCCWGTGTCAC-BHQ1 3′	[16]
*Rickettsia* spp.	*ompA*	Conventional PCR	5′ ATGGCGAATATTTCTCCAAAA 3′ 5′ GTTCCGTTAATGGCAGCATCT 3′	[17]
*Apicomplexa*	*18S rRNA*	Conventional PCR	5′ GTCTTGTAATTGGAATGATGG 3′ 5′ TAGTTTATGGTTAGGACTACG 3′	[18]

**Table 2:** Tick species and detected tick-borne pathogens in different wild hosts in Greece.

	Number of Ticks					Number of Ticks Infected with Pathogens (%)							
Host	*Hyalomma aegyptium*	*Hyalomma marginatum*	*Hyalomma anatolicum*	*Ixodes frontalis*	*Ixodes ventalloi*	*Rickettsia* spp.		*Hemolivia* *mauritanica*	*Ehrlichia* spp./*Anaplasma* spp.	*Babesia* spp.	*Bartonella* spp.	*CCHF*	*TBE*
						*R. aeschlimannii*	*R. africae*						
*Testudo marginata*	46	-	-	-	-	5 (11)	1 (2)	4 (9)	N/A	N/A	N/A	N/A	N/A
*Tyto alba*	-	18	-	-	-	11 (61)	N/A	N/A	N/A	N/A	N/A	N/A	N/A
*Columba oenas*	1	-	-	-	-	1 (100)	N/A	N/A	N/A	N/A	N/A	N/A	N/A
*Erinaceus europaeus*	2	-	-	-	-	2 (100)	N/A	N/A	N/A	N/A	N/A	N/A	N/A
*Athene noctua*	1	-	-	-	-	N/A	N/A	N/A	N/A	N/A	N/A	N/A	N/A
*Testudo hermanni*	2	-	-	-	-	2 (100)	N/A	N/A	N/A	N/A	N/A	N/A	N/A
*Buteo buteo*	6	-	-	-	-	N/A	N/A	N/A	N/A	N/A	N/A	N/A	N/A
*Pica pica*	-	-	-	1	-	N/A	N/A	N/A	N/A	N/A	N/A	N/A	N/A
*Canis vulpes*	-	-	-	-	1	N/A	N/A	N/A	N/A	N/A	N/A	N/A	N/A
*Lepus europaeus*	-	-	3	-	-	N/A	N/A	N/A	N/A	N/A	N/A	N/A	N/A

## Data Availability

The original contributions presented in this study are included in the article. Further inquiries can be directed to the corresponding author.
